# Un cas de fracture luxation négligée du coude avec conservation de la fonction du coude

**DOI:** 10.11604/pamj.2015.20.199.4265

**Published:** 2015-03-03

**Authors:** Kamal Lahrach, Oussama Ammoumri, Amine Mezzani, Mounir Benabid, Amine Marzouki, Fawzi Boutayeb

**Affiliations:** 1 Service de Chirurgie Orthopédique et Traumatologique (A), Centre Hospitalier Universitaire Hassan-II, Faculté de Médecine et de Pharmacie, Fès, Maroc

**Keywords:** Conservation fonction, coude, luxation-négligée, Conservation fonction, elbow, neglected dislocation

## Abstract

Les fractures luxations du coude sont rares et souvent mal tolérées chez les sujets jeunes actifs. Nous rapportons un cas de fracture-luxation du coude remontant à 20 ans. C'est un jeune de 35 ans, victime il y a 20 ans d'un traumatisme fermé, suite à une chute lors d'un match du football, de son coude gauche occasionnant une fracture-luxation du coude. Le patient a refusé une intervention chirurgicale avec une auto-rééducation. L'examen a mis en évidence une conservation de la fonction du coude. Un bilan radiologique a montré une fracture luxation du coude avec remaniement de la palette humérale. Une abstention thérapeutique a été décidée devant l'ancienneté de la fracture-luxation et la gêne fonctionnelle minime engendrée. Contrairement aux autres séries, la fracture-luxation dans notre cas était bien tolérée malgré le jeune âge du patient.

## Introduction

Les fractures négligées du coude sont peu fréquentes dans les pays industrialisés. Elles ne sont pas rares en revanche dans notre contexte. Le pronostic est marqué par une raideur même après une intervention chirurgicale. Les auteurs rapportent le cas d'une fracture luxation du coude négligée avec une bonne conservation de la fonction du coude.

## Patient et observation

**Figure 1:**
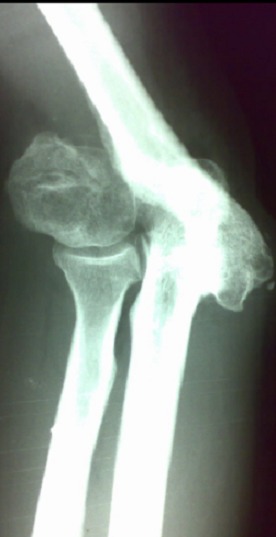
Radiographie de face montrant une fracture luxation du coude avec cal vicieux du condyle huméral externe

**Figure 2:**
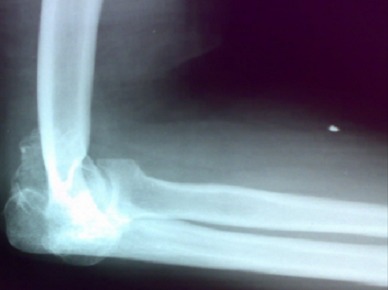
Radiographie de profil montrant la radio-ulnaire en place

**Figure 3:**
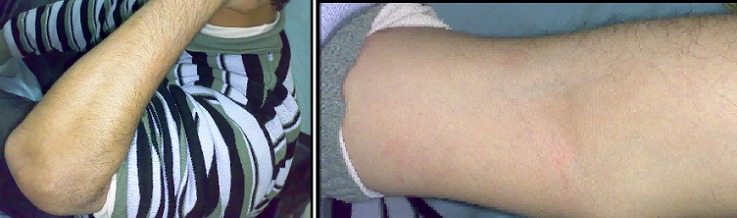
Extension-flexion du coude sont conservée avec une bonne trophicité musculaire

Mr H.R. âgé de 35 ans, maçon de profession victime il y a 20 ans, lors d'un match de football, d'une chute avec réception sur le coude occasionnant chez lui un traumatisme fermé du coude gauche. Le patient a consulté aux urgences mais il a refusé une intervention chirurgicale, avec une auto-rééducation. L’évolution a été marquée par la disparition progressive de la douleur et la reprise de la mobilité. Le patient a développé cinq ans après des névralgies dans le territoire du nerf cubital traitées par une neurolyse avec transposition du nerf cubital (le patient n'a pas fait de séances de rééducation). À l'occasion d'un contrôle, une radiographie du coude face et profil, a objectivé une fracture-luxation du coude avec un cal vicieux du condyle humérale externe (Figure 1, Figure 2). L'examen clinique trouve un coude bien axé; une saillie interne en regard de l’épitrochlée, une légère laxité interne sans déficit vasclo-nerveux. Flexion-extension: 0°/0°/140° et la Prono-suppination: 90°/0°/90° avec une bonne trophicité musculaire (Figure 3), l'examen nerveux de la main est normal. Devant l'ancienneté de la luxation et la gêne fonctionnelle minime engendrée, une abstention thérapeutique a été décidée.

## Discussion

La fracture-luxation négligée du coude est une entité clinique qui se voit encore dans les pays où les structures sanitaires n'ont pas encore atteint leur développement. Elle est responsable le plus souvent d'un enraidissement. Martini [1] classe les raideurs du coude en deux groupes: les raideurs fonctionnelles où la flexion du coude atteint généralement 80° à 90° avec une bonne adaptation de l’épaule et de la main, la mobilité du coude permet des gestes utiles (porter la main à la bouche et aux cheveux, l'attache du soutien-gorge chez la femme) et les raideurs non fonctionnelles où la flexion du coude ne dépasse pas 70° et ne permettant pas les gestes utiles malgré les efforts d'adaptation de l’épaule et de la main. La prise en charge des fractures luxations négligées du coude est variable dans la littérature. Speed [2], Cambell [3] et Vangonder [4] sont les premiers à rapporter les résultats de leur courte expérience de l'affection et de son traitement. Les auteurs s'abstiennent définitivement devant une raideur à la limite de l'adaptation fonctionnelle et qui peut s'améliorer après quelques semaines de rééducation. Certains auteurs (Bourrel [5], Krisham [6]) et en particulier Martini [1] ont souligné l'adaptation du coude luxé avec le temps. Martini [1] n'opère pas systématiquement les luxations négligées du coude. La réduction sanglante reste l'indication fréquente mais non systématique. Plusieurs techniques chirurgicales sont rapportées dans la littérature. Dishino [7] traitait 23 luxations négligées du coude par résection de la palette humérale. Cette technique donne de bons résultats sur la mobilité mais elle a comme inconvénient un risque de laxité et d'instabilité. Dans une étude plus récente, Dishino [8] rapporte une série de 81 cas opérés dont 52 revus avec un recul compris entre quatre mois et sept ans, l'auteur réalise 40 fois la résection de la palette humérale et 41 fois la reposition. Les résultats sur la mobilité sont superposables pour les deux techniques. La résection semble être supérieure pour la pronosupination mais c'est une technique mutilante qui présente un risque de laxité et d'instabilité. Dans notre cas, nous avons préconisé une abstention thérapeutique vue l'ancienneté de la fracture-luxation d'une part et l'absence de gêne fonctionnelle d'autre part.

## Conclusion

Les fractures luxations du coude sont des lésions graves souvent mal tolérées et de prise en charge difficile. Nous insistons sur la nécessité de l'amélioration des structures sanitaires dans notre pays pour prévenir ce type de lésion de prise en charge facile si elles sont diagnostiquées en urgence.
